# Ephraim McDowell, M.D.

**Published:** 1878-08-20

**Authors:** 


### EPHRAIM McDOWELL, M.D.

A monument is soon to be erected in honor of Dr. McDowell as the father of ovariotomy. It will be a shaft of granite, 30 feet high, with an appropriate inscription upon it.

